# 2308. A Nationwide Surveillance of the Clinical Presentation and Genomic Characteristics of *Listeria monocytogenes* Infections in Japan; Preliminary data

**DOI:** 10.1093/ofid/ofac492.140

**Published:** 2022-12-15

**Authors:** Koh Shinohara, Yasuhiro Tsuchido, Satomi Yukawa, Taro Noguchi, Masaki Yamamoto, Yasufumi Matsumura, Miki Nagao

**Affiliations:** Kyoto University Graduate School of Medicine, Kyoto, Kyoto, Japan; Kyoto University Graduate School of Medicine, Kyoto, Kyoto, Japan; Kyoto University Graduate School of Medicine, Kyoto, Kyoto, Japan; Kyoto University Graduate School of Medicine, Kyoto, Kyoto, Japan; Kyoto University Graduate School of Medicine, Kyoto, Kyoto, Japan; Kyoto University Graduate School of Medicine, Kyoto, Kyoto, Japan; Kyoto University Graduate School of Medicine, Kyoto, Kyoto, Japan

## Abstract

**Background:**

*Listeria monocytogenes* is the cause of listeriosis and is associated with foodborne outbreak and severe diseases in immunocompromised persons. However, the clinical and genomic characteristics of listeriosis remains unclear in Japan. We describe preliminary findings from a nationwide surveillance of patients and isolates of listeriosis in Japan over 11-year period.

**Methods:**

We conducted a nationwide, multi-center retrospective study of consecutive cases with a confirmed diagnosis of listeriosis between 2011 and 2021. 25 hospitals participated in this study. Clinical characteristics, treatment and outcome were retrieved from the medical records. The preserved *L. monocytogenes* isolates were investigated with molecular analysis using whole genome sequencing.

**Results:**

As of April 2022, a total of 113 cases from 16 hospitals and 26 isolates from 7 hospitals were included in this study. Sixty-one (54%) were male and median age was 74.0 (interquartile range: 64.0 – 82.0). Ten (9%) perinatal infections, 66 (58%) cases of bacteremia, 29 (26%) cases of neurolisteriosis, and eight (7%) other infections were included. In eight episodes with ten perinatal cases, four (50%) episodes experienced fetal loss or neonatal deaths. In 103 non-perinatal cases, 56 (54%) cases received immunosuppressive therapy and in-hospital mortality rate was 17.0%. In 29 neurolisteriosis cases, 13 (45%) cases were associated with poor outcome (mortality or neurological sequelae). In 26 isolates, from two perinatal infections, 13 bacteremia, 9 neurolisteriosis and two other infections, clonal complex (CC) 1, CC2 and CC3 were predominant and accounted for 18 (69%) isolates (Figure). CC1 (5/8 isolates) and CC3 (2/6 isolates) were predominantly found in neurolisteriosis. Variations of the presence of several virulence factors associated with adherence, invasion, and exotoxin were observed. CC1 and CC2 lacked ActA and Ami, whereas Listeriolysin S (LLS) were seen in CC1, CC3 and CC4.

**Conclusion:**

In Japan, most cases of listeriosis are sporadically seen in elderly patients with co-morbidities, instead of foodborne outbreak. Outcomes of neurolisteriosis and perinatal listeriosis were poor. CC1 and CC3, which harbored LLS, were associated with neurolisteriosis.

**Disclosures:**

**All Authors**: No reported disclosures.

